# Microsatellite Instability Status and the Expression of p16 and Cyclin D1 Proteins in Uterine Adenosarcoma and Their Clinicopathological Significance

**DOI:** 10.5146/tjpath.2022.01580

**Published:** 2023-01-15

**Authors:** Alev Ok Atılgan, Eda Yılmaz Akcay, Ozlem Ozen, A. Nihan Haberal Reyhan, Ali Ayhan

**Affiliations:** Department of Pathology, Baskent University, Faculty of Medicine, Ankara, Turkey; Department of Obstetrics and Gynecology, Division of Gynecologic Oncology, Baskent University, Faculty of Medicine, Ankara, Turke

**Keywords:** Adenosarcoma, Microsatellite instability, p16, Cyclin D1

## Abstract

***Objective:*** Uterine adenosarcoma has low malignant potential, except in cases with sarcomatous overgrowth (SOG) and a high-grade morphology. We here point out the prognostic clinicopathological and immunohistochemical features as well as the microsatellite instability (MSI) status of high- and low-grade adenosarcomas.

***Material and Method:*** In this study, DNA mismatch repair proteins, p16, cyclin D1, ER, PR, and CD10 were examined in uterine adenosarcoma cases using immunohistochemistry. The association between these proteins and clinicopathological parameters was also evaluated.

***Results:*** ER, PR and CD10 expressions were lower and weaker in high-grade adenosarcomas with SOG compared to low-grade adenosarcomas without SOG (p < 0.05). p16 positivity was more frequent in high-grade adenosarcomas than low-grade adenosarcomas (p < 0.05). There was no statistically significant difference between cyclin D1 positivity, MSI, and other clinicopathological parameters (p ≥ 0.05). Cyclin D1 positivity and loss of CD10 expression were associated with shorter disease-free survival (DFS). Loss of ER and CD10 expression was associated with shorter overall survival (OS) (p < 0.05). MSI was not associated with DFS or OS (p ≥ 0.05).

***Conclusion:*** These results suggested that p16 positivity, and loss of ER, PR, and CD10 expression were predictors of high-grade morphology. Additionally, the current study showed that cyclin D1-positive tumors had high recurrence rates; however, no significant relationships were found between MSI and DFS or OS in patients with uterine adenosarcoma. Further investigations are required to determine the importance of p16, cyclin D1, and MSI in uterine adenosarcomas.

## INTRODUCTION

Uterine adenosarcoma, a rare mixed epithelial and mesenchymal tumor of the female genital tract, accounts for 5% and 10% of all uterine sarcomas (1). These tumors typically comprise benign epithelial elements and low-grade malignant mesenchymal components. The tumor exhibits a biphasic appearance consisting of tubular, dilated cleft-like glands lined by a benign-appearing epithelium and malignant cellular stroma (2,3). Stroma is typically a low-grade sarcoma that usually has no specific line of differentiation, although some consider it to resemble endometrial stromal sarcoma (2,3). The presence of sarcoma without any epithelial component in > 25% of the tumor refers to sarcomatous overgrowth (SOG). It is generally associated with deeper myometrial involvement, lymphovascular invasion, and worse prognosis and recurrence (2–4).

In the literature, it has been shown that SOG tends to be associated with a high-grade morphology. However, it should be kept in mind that there may be minor high-grade morphology in the tumor without SOG. In addition, SOG can also be seen in purely low-grade adenosarcomas (5). Almost all studies about adenosarcomas have focused on the presence of SOG in the literature. A few studies have distinguished between low-grade adenosarcoma and high-grade adenosarcoma (5–7). Soslow and Longacre have proposed that high-grade adenosarcomas have an aggressive course (8). It has been thought that high-grade morphology may be an independent factor separate from SOG (5). Hodgson et al. also showed that high-grade adenosarcomas have distinctive molecular characteristics along with morphologic and clinic features from low-grade adenosarcomas (5). Although the latest WHO 2020 classification of female genital tract tumors does not yet include the grading of uterine adenosarcomas, the College of American Pathologists (CAP, 2018) protocol recommends recording in the pathology report whether the stromal component is morphologically “low-grade” or “high-grade” for adenosarcomas without sarcomatous overgrowth. A high-grade morphology is defined as sarcoma with severe nuclear atypia and pleomorphism identifiable at low power magnification, characterized by enlarged ovoid or spindle nuclei with coarse chromatin and prominent nucleoli (5). However, there is not yet a cut-off value on nuclear size and mitotic count in the distinction between high- and low-grade morphology.

The deoxyribonucleic acid (DNA) mismatch repair (MMR) system protects the human genome from intrinsic and extrinsic factors via short repeating motifs in the DNA called microsatellites, which repair mismatching errors such as inappropriate nucleotide insertions and deletions as well as single nucleotides. When these errors are not corrected, genomic stability is disrupted during DNA replication and recombination (9). Deficient MMR (dMMR) is a major cause of genomic instability and results in the accumulation of numerous mutations in microsatellite sequences, resulting in microsatellite instability (MSI) (9,10). The primary DNA MMR proteins associated with MSI by inactivation are MutL protein homolog 1 (MLH1), MutS protein homolog 2 (MSH2), MutS protein homolog 6 (MSH6), and postmeiotic segregation increased 2 (PMS2). These proteins interact as heterodimers, i.e., MSH2 couples with MSH6, and MLH1 couples with PMS2. MSI has been most closely studied in colorectal cancers; however, it has been found in various cancer types, including gynecological tumors (9,11). In the context of uterine adenosarcomas, MSI and dMMR are not yet fully understood, and their clinical significance as prognostic factors has not yet been explored.

The p16 protein plays a role as a tumor suppressor that negatively regulates the cell cycle by inhibiting the activity of cyclin D-dependent kinases to prevent phosphorylation of the RB family protein and cyclin D1 bind to cdk4, leading to the inactivation of RB genes (12,13). Gene mutation, deletion, or epigenetic silencing, or in cases where overexpression of cyclin D1 occurs, can lead to RB or p16 inactivation and abnormal cell proliferation (13). Various cancers involving the mutation or overexpression of p16 and cyclin D1 have been identified (13–15).

The aim of this study was twofold. First, p16, cyclin D1, ER, PR, and CD10 expression in uterine adenosarcomas was evaluated to determine the possible impacts on the prognosis. Second, we investigated the frequency and the prognostic effect of MSI by evaluating the immunohistochemical expression of MMR proteins in uterine adenosarcoma.

## MATERIAL and METHODS

### Patient Selection

Twenty cases of uterine adenosarcoma, diagnosed between January 1, 2009 and December 30, 2020, were included in this study. The research was approved by the Ethics Committee of the Faculty of Medicine (KA21/419), and all protocols conformed to the ethical guidelines of the 1975 Helsinki Declaration. The hospital records of the included patients were reviewed, and the clinical follow-up findings were noted. The diagnosis of adenosarcoma and SOG were based on the standard criteria adopted by the World Health Organization (2020). Each tumor was classified as either low or high grade. A high-grade morphology was defined as sarcoma with severe nuclear atypia and pleomorphism identifiable at low power magnification, characterized by enlarged ovoid or spindle nuclei with coarse chromatin and prominent nucleoli (5). The tumor stage of all patients was noted according to the International Federation of Gynecology and Obstetrics (FIGO Cancer Report 2018) staging system.

### Tissue Microarray and Immunohistochemistry

Two representative foci involving different areas (2 mm in diameter) supporting the diagnosis and showing both epithelial and stromal areas including high-grade or low-grade morphology were punched from the original and inserted into a new paraffin block. Serial-sectioned slides were obtained with a conventional microtome of 4-μm-thickness for immunohistochemistry using the following primary antibodies: estrogen receptor (ER), progesterone receptor (PR), CD10, cyclin D1, p16, CD117, MLH-1, PMS-2, MSH-2, and MSH-6. We used a Dako Omnis (Agilent, Santa Clara, CA) system and EnVision FLEX staining kits to perform immunohistochemical staining. Sections were maintained at 60°C for 60 min and dewaxed using a Clearify (Dako) solution at 25°C for 1 min in an autostainer. Heat-induced antigen retrieval was performed using an ethylenediaminetetraacetic acid/citrate buffer (EnVision FLEX HRS, high pH) at 97°C for 60 min for antibodies (EnVision FLEX HRS). The sections were rinsed with a wash buffer, and sections were incubated with anti-MLH1 (clone ES05, mouse), PMS2 (clone EP51, rabbit), MSH2 (clone FE11, mouse), and MSH6 (clone EP49, rabbit), ER (clone EP1, rabbit), PR (clone PgR636, mouse), CD10 (clone 56C6, mouse), cyclin D1 (clone EP12, rabbit) (all Ready-to-Use, all from DAKO), and p16 (clone Y123261, Mouse, ready-to-use from ABM). Sections were then incubated with a peroxidase solution (EnVision FLEX peroxidase-blocking reagent; Dako) for 3 min. These were rinsed, reactivated in an EnVision FLEX/horseradish peroxidase solution for 20 min, incubated for 5 min with an EnVision FLEX substrate working solution (DAKO) for visualization, and then counterstained with hematoxylin. Appropriate positive and negative controls were used.

### Analysis of Immunohistochemical Staining

**Figure 1:**
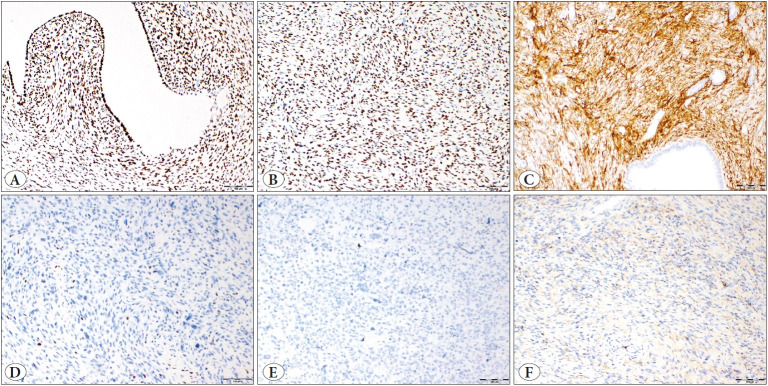
Immunohistochemical staining of ER, PR, CD10. **A)** ER, **B)** PR, **C)** CD10 strong positivity. **D)** ER, **E)** PR, **F)** CD10 negativity (x100 original magnification).

**Figure 2:**
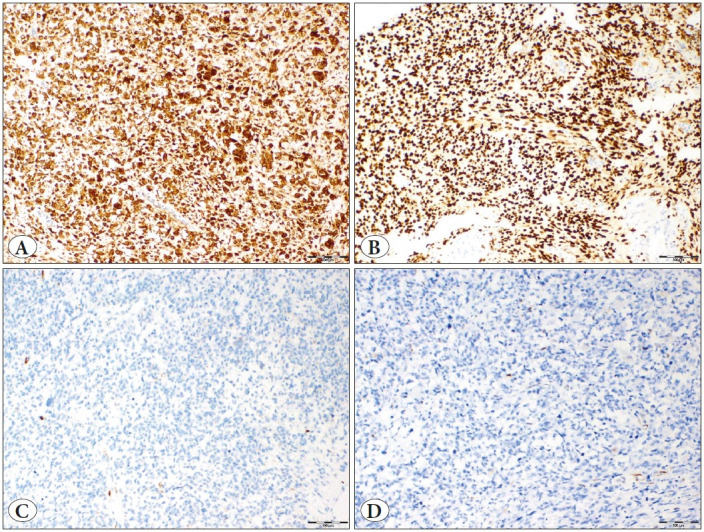
Immunohistochemical staining of p16, cyclin D1. **A)** p16, **B)** cyclin D1 strong positivity. **C)** p16, **D)** cyclin D1 negativity (x100 original magnification).

**Figure 3:**
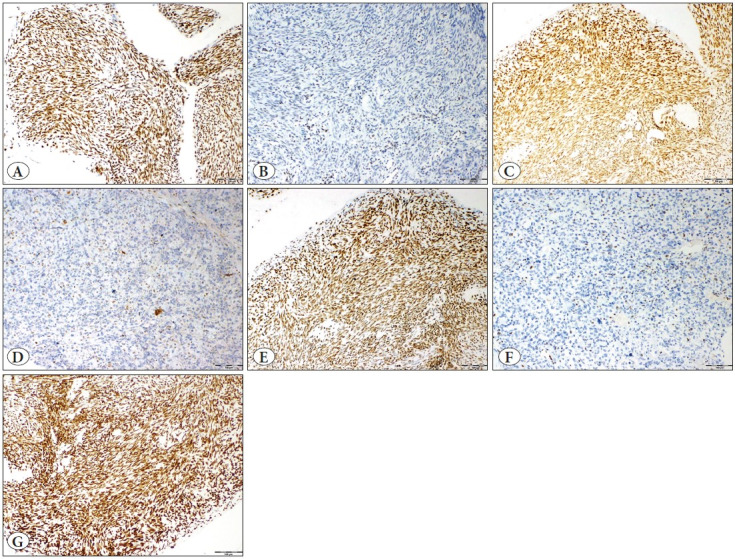
Immunohistochemical staining of MMR proteins **A)** retained MLH1 protein, **B)** loss of MLH1 protein, **C)** retained PMS2 protein, **D)** loss of PMS2 protein, **E)** retained MSH2 protein, **F)** loss of MSH2 protein, **G)** retained MSH6 protein (x100 original magnification).

Two researchers (AOA, EYA) independently scored the immunohistochemistry-stained slides using a double-headed microscope without prior knowledge of any relevant clinicopathological information. Staining was analyzed only when each core included ≥ 50% tumor tissue. For ER, PR, and CD10, the intensity was scored as negative (0), weak (1+), moderate (2+), or strong (3+) and the percentage of positive tumor cells was scored from 0 to 100%. The intensity score was multiplied by the percentage and the *H*-score was used as the final semi-quantitative score for each case (6). The immunostaining was considered mild when *H*-score was ≤ 100, moderate when 101-200, and strong when 201-300 (Figure 1). Tumors showing ≥ 70% moderate to strong nuclear staining for cyclin D1 and p16 were considered as positive (16) (Figure 2). We assessed loss of immunohistochemical expression of MMR proteins in tumor cells in the presence of positive internal control. Tumors with intact immunohistochemical expression of all four MMR proteins were considered microsatellite stable (MSS), whereas tumors with loss of immunohistochemical expression of one or more MMR proteins were considered to show MSI (Figure 3). Membrane staining was interpreted as indicating CD117 positivity.

### Statistical Analysis

Statistical analyses were performed using the Statistical Package for the Social Sciences version 26.0 (SPSS Inc., Chicago, IL, USA). The variables were investigated using analytical methods (Shapiro-Wilk test) to determine whether or not they were normally distributed. Descriptive analyses were presented using median (minimum (min)-Maximum (max)) for numerical variables. Since the variables did not show a normal distribution, non-parametric tests were performed. Comparison of numerical data was performed using the nonparametric Mann–Whitney test and Kruskal-Wallis test. Qualitative variables were examined using the Fisher’s exact test. Disease-free survival (DFS) and overall survival (OS) rates were estimated using the Kaplan-Meier method and were compared using the long-rank test. A p-value of < 0.05 was considered statistically significant.

## RESULTS

### Clinicopathological Features

The median (min-max) age at the time of diagnosis was 55 (15-78) years. Among the 20 tumors, 17 (85%) arose from the endometrium (3 from the lower uterine segment), 1 (5%) from the adenomyotic foci of the myometrium, and 2 (10%) from the cervix. The tumors ranged from 2 to 15 cm in diameter (median 7.3 cm). At the time of diagnosis, 4 (20%) of the tumors were FIGO stage Ia, 12 (60%) stage Ib, 3 (15%) stage Ic, and 1 (5%) stage IV disease. FIGO stage Ia and Ib were accepted as early stages, while stages Ic and IV were considered advanced stages.

Sixteen tumors showed myometrial or cervical invasion. Two tumors arose from the endometrium, and one tumor had no myometrial or cervical stromal invasion arising from the cervix uteri. One tumor was limited to the adenomyotic focus arising from the myometrium.

Among the 20 tumors, 11 (55%) had a high-grade component (all of which had SOG), and 9 (45%) tumors were purely low grade (none had SOG) (p≤0.001). Seven tumors had heterologous elements, including chondrosarcomatous differentiation in 1 case, rhabdomyosarcomatous differentiation in 5, and benign cartilage differentiation in 1. All of the tumors containing malignant heterologous elements were high grade (p=0.045). The median (min-max) mitotic count was 8 (3-13) per 10 high powered fields (HPFs) in the high-grade adenosarcoma group vs. 3 (2-6)/10 HPFs in the low-grade adenosarcoma group (p=0.002). Of the 20 tumors, 4 (20%, all high grade) had lymphovascular invasion (p=0.045). One (5%) of the tumors that had lymph node metastases showed thoracal vertebra and lung metastases at the time of diagnosis. None of the tumors had omental metastases and positive peritoneal cytology.

There was no statistically significant difference between the high- and low-grade adenosarcoma groups in terms of age at diagnosis, tumor size, presence/depth of myometrial invasion, or FIGO stage (p≥0.05).

### The Association of ER, PR, CD10 Expressions with Clinicopathological Characteristics

ER, PR and CD10 expressions were lower and weaker in high-grade adenosarcomas with SOG compared to low-grade adenosarcomas without SOG (p=0.022, p=0.017, p≤0.001, respectively). ER and PR expressions were lower and weaker in adenosarcomas containing heterologous elements than in those without heterologous elements (p=0.005, p=0.044, respectively). There was no statistically significant association between ER, PR and CD10 expressions and other clinicopathological parameters (p≥0.05) (Table 1).

**Table 1:** The association between ER/PR/CD10 expression and clinicopathologic features.

**Clinicopathologic variables**		**ER Expression**				**PR Expression**				**CD10 Expression**		
		**Strong**		**Moderate-Mild**	**p**	**Strong**	**Moderate-Mild**		**p**	**Strong**	**Moderate-Mild**	**p**
	**n (%)**	**9 (45)**		**11 (55)**		**7 (35)**	**13 (65)**			**10 (50)**	**10 (50)**	
**Grade**												
High grade	11 (55)	2 (18.2)		9 (81.8)	0.022*	1 (9.1)	10 (90.9)		0.017*	1 (9.1)	10 (90.9)	≤ 0.001*
Low grade	9 (45)	7 (77.8)		2 (18.2)		6 (66.7)	3 (33.3)			9 (100)	0 (0)	
**Sarcomatous overgrowth**												
Present	11 (55)	2 (18.2)		9 (81.8)	0.022*	1 (9.1)	10 (90.9)		0.017*	1 (9.1)	10 (90.9)	≤ 0.001*
Absent	9 (45)	7 (77.8)		2 (18.2)		6 (66.7)	3 (33.3)			9 (100)	0 (0)	
**Heterologous elements**												
Present	7 (35)	0 (0)		7 (100)	0.005*	0 (0)	7 (100)		0.044*	2(28.6)	5 (71.4)	0.350
Absent	13 (65)	9 (69.2)		4 (30.8)		7 (53.8)	6 (46.2)			8 (61.5)	5 (38.5)	
**Lymphovascular invasion**												
Present	4 (20)	0 (0)		4 (100)	0.094	0 (0)	4 (100)		0.249	0 (0)	4 (100)	0.087
Absent	16 (80)	9 (56.3)		7 (43.7)		7 (43.8)	9 (56.3)			10 (62.5)	6 (37.5)	
**Myometrial or cervical stromal invasion**												
<50%	17(85.5)		8 (47.1)	9 (52.9)	1	6 (35.3)	11 (64.7)		1	9 (52.9)	8 (47.1)	1
≥50%	3 (15.5)		1 (33.3)	2 (66.7)		1 (33.3)	2 (66.7)			1 (33.3)	2 (66.7)	
**FIGO Stage**												
Stage Ia-Ib	16 (80)		8 (50)	8 (50)	0.591	7 (43.8)	9 (56.2)		0.619	9 (56.2)	7 (43.8)	0.582
Stage Ic-IVb	4 (20)		1 (25)	3 (75)		1 (25)	3 (75)			1 (25)	3 (75)	
**Recurrence**												
No Recurrence	16 (80)		8 (50)	8 (50)	0.591	7 (43.8)	9 (56.3)		0.249*	10 (62.5)	6 (37.5)	0.087
Recurrence	4 (20)		1 (25)	3 (75)		0 (0)	4 (100)			0 (0)	4 (100)	

* Statistically significant

### The Association of p16, cyclin D1, and CD117 Expression with Clinicopathological Characteristics

All high-grade adenosarcomas had p16 positivity, and 5 (55.6%) of 9 low-grade adenosarcomas exhibited p16 positivity (p=0.026). p16 positivity was more frequent in adenosarcomas containing SOG than in those without SOG. (p=0.026). There was no statistically significant association between p16 positivity and other clinicopathological parameters (p≥0.05) (Table 2).

**Table 2:** The association between p16/cyclin D1 expression, Microsatellite Instability Status and clinicopathologic features.

**Clinicopathologic variables**			**p16 Expression**				**Cyclin D1 Expression**					**Microsatellite Instability Status**		
			**Positive**		**Negative**	***p***	**Positive**	**Negative**		***p***		**MSI**	**MSS**	***p***
	**n (%)**		**16 (80)**		**4 (20)**		**8 (40)**	**12 (60)**				**4 (20)**	**16 (80)**	
**Grade**														
High grade	11 (55)		11 (100)		0 (0)	0.026*	5 (45.5)	6 (54.5)		0.670		2 (18.2)	9 (81.8)	1
Low grade	9 (45)		5 (55.6)		4 (44.4)		3 (33.3)	6 (66.7)				2 (22.2)	7 (77.8)	
**Sarcomatous overgrowth**														
Present	11 (55)		11 (100)		0 (0)	0.026*	5 (45.5)	6 (54.5)		0.670		2 (18.2)	9 (81.8)	1
Absent	9 (45)		5 (55.6)		4 (44.4)		3 (33.3)	6 (66.7)				2 (22.2)	7 (77.8)	
**Heterologous elements**														
Present	7 (35)		6 (85.7)		1 (14.3)	1	3 (42.9)	4 (57.1)		1		2(28.6)	5 (71.4)	0.587
Absent	13 (65)		10 (76.9)		1 (23.1)		5 (38.5)	8 (61.5)				2 (15.4)	11 (84.6)	
**Lymphovascular invasion**														
Present	4 (20)		4 (100)		0 (0)	0.538	1 (25)	3 (75)		0.619		2 (50)	2 (50)	0.162
Absent	16 (80)		12 (75)		4 (25)		7 (43.8)	9 (56.3)				2 (12.5)	14 (87.5)	
**Myometrial or cervical stromal invasion**														
<50%		17(85.5)		14 (82.4)	3 (17.6)	0.509	6 (35.3)	11 (64.7)		0.537		4 (23.5)	13 (76.5)	1
≥50%		3 (15.5)		2 (66.7)	1 (33.3)		2 (66.7)	1 (33.3)				0 (0)	3 (100)	
**FIGO Stage**														
Stage Ia-Ib		16 (80)		13 (81.3)	3 (18.7)	1	6 (37.55)	10 (62.5)		1		10 (62.5)	6 (37.5)	1
Stage Ic-IVb		4 (20)		3 (75)	1 (25)		2 (50)	2 (50)				3 (75)	1 (25)	
**Recurrence**														
No Recurrence		16 (80)		12 (75)	4 (25)	0.538	4 (25)	12 (75)		0.01*		4 (25)	12 (75)	0.538
Recurrence		4 (20)		4 (100)	0 (0)		4 (100)	0 (0)				0 (0)	4 (100)	

**MSI:** Indicates Microsatellite instable, **MSS:** Microsatellite stable. * Statistically significant

Five (45.5%) of the 11 high-grade adenosarcomas and 3 (33.3%) of the 9 low-grade adenosarcomas showed cyclin D1 positivity. However, there was no significant association between cyclin D1 positivity and tumor grade (p=0.670). There was no statistically significant association between cyclin D1 positivity and other clinicopathological parameters (p≥0.05) (Table 2).

Neither high grade nor low-grade adenosarcoma exhibited CD117 immunoreactivity.

### The Association of MMR Protein Expression with Clinicopathologic Characteristics

The loss of MLH1, PMS2, MSH2, and MSH6 expression was detected in 4 (20%), 1 (5%), 1 (5%) and 0 (0%) cases, respectively. Out of 20 adenosarcomas, 4 (20%) presented a loss of expression for at least one MMR protein; 1 (5%) showed a loss of three proteins (MLH1, PMS2, MSH2), 3 (15%) showed a loss of one MMR protein (MLH1). The remaining 16 tumors (80%) were positive for four MMR proteins. As a result, 16 tumors that expressed all MMR proteins were accepted as MSS, and four tumors that showed clonal loss of at least one of the MMR proteins were accepted as MSI. Accordingly, the frequency of MSI in adenosarcoma was 20% in our study group (Figure 3). None of the patients underwent the MLH1 methylation test and genetic consultation. None of the patients had a known history of Lynch syndrome.

Two (18.2%) of 11 high-grade adenosarcomas and two (22.2%) of 9 low-grade adenosarcomas were MSI. There was no statistically significant association between MSI and tumor grade and other clinicopathologic parameters (p≥0.05) (Table 2).

### Survival Analysis

#### Disease-Free Survival

The median (min-max) time for recurrence was 9.6 (5.7-19.4) months. Only four (20%) patients showed recurrence occurring in the upper abdominal region and vaginal cuff. All of the tumors (100%) that showed recurrence were high-grade, and 9 of 16 (56.3%) nonrecurring tumors were low-grade. Univariate Kaplan–Meier/ Log-rank analyses revealed that high-grade adenosarcomas and tumors with SOG tended to show a higher incidence of disease recurrence (p=0.03). While the median (min-max) DFS time of patients with advanced FIGO stage was 7.2 (3.9-140.8) months, for patients with early FIGO stage this was 57.0 (2.5-102.0) months. Adenosarcomas with advanced FIGO stage had significantly decreased DFS than those with early FIGO stage (p=0.001). Adenosarcomas with higher mitoses had significantly decreased DFS than those with lower mitoses (p≤0.001). Adenosarcomas with deeper myometrial invasion also had significantly decreased DFS than those with superficial or any myometrial invasion (p=0.029). However, the presence of lymphovascular invasion, heterologous elements did not affect the DFS rate (p≥0.05).

**Figure 4:**
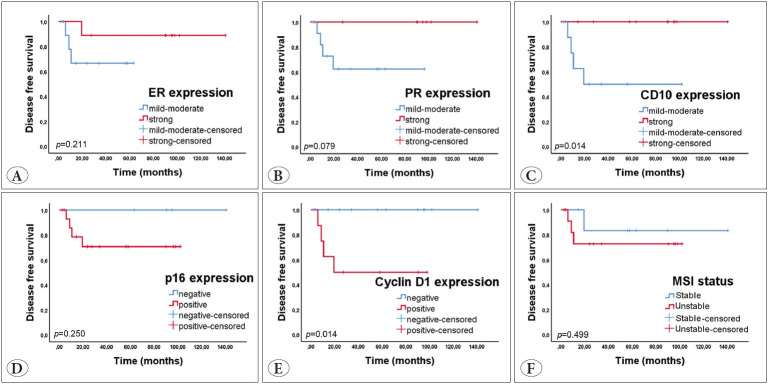
The Kaplan-Meier curves of **A)** ER, **B)** PR, **C)** CD10, **D)** p16, **E)** cyclin D1, **F)** MSI status for disease-free survival. Patients with loss of CD10 expression and cyclin D1 positivity had a significantly shorter disease-free survival.

ER, PR, and CD10 expression of the tumors showed that recurrence was lower and weaker than in those without recurrence. We found that CD10 expression, not ER and PR expression, affected the DFS rate (p=0.014). Cyclin D1 positivity tended to show a higher incidence of disease recurrence (p=0.014). All tumors that had recurrence showed p16 positivity. However, p16 positivity had no impact on the DFS rate (p≥0.05). Four of 16 patients with MSS adenosarcoma showed recurrence. None of the patients with MSI adenosarcoma showed recurrence. However, MSI status did not affect the DFS rate (p≥0.05) (Figure 4).

#### Overall Survival

The median (min-max) follow-up time was 60.6 (2.5-140) months. Four (20%) patients died because of their disease. Two (10%) patients died due to cardiovascular deficiency without evidence of uterine adenosarcoma recurrence and were considered censored. Two (10%) patients were alive and still living with the disease, and 12 (60%) patients were alive with no evidence of disease.

All the patients who died of disease were high-grade, and all the patients with low-grade tumors were alive. The median (min-max) OS of patients with high-grade adenosarcoma was 34.1 (2.5-102.0) months, and for patients with low-grade adenosarcoma it was 90.5 (14.3-140.8) months. Univariate Kaplan–Meier/ Log-rank analyses revealed that high-grade adenosarcomas and tumors with SOG statistically showed marginal significance (p=0.05). While the median (min-max) OS time of patients with advanced FIGO stage was 43.1 (3.9-140.8) months, for patients with early FIGO stage this was 60.7 (2.5-102.0) months. Advanced FIGO stage, presence of lymphovascular invasion, and higher mitotic count and heterologous elements were significantly associated with decreased OS rates (p=0.025, p≤0.001, p ≤.001, p = 0.038, respectively). Myometrial invasion was not significantly associated with the OS rate (p=0.189).

**Figure 5:**
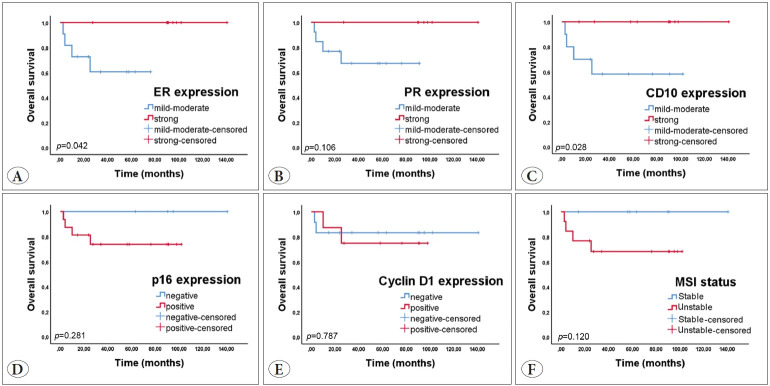
The Kaplan-Meier curves of **A)** ER, **B)** PR, **C)** CD10, **D)** p16, **E)** cyclin D1, **F)** MSI status for overall survival. Patients with loss of ER/CD10 expression had a significantly shorter overall survival.

The loss of ER and CD10 immunoreactivity was associated with a decreased OS rate (p=0.042, p=0.028, respectively). While PR, p16, and cyclin D1 immunoreactivity were not significantly associated with the OS rate (p≥0.05). Three of 16 patients with MSS adenosarcoma died, and one of 4 patients with MSI adenosarcoma died; however, the MSI status did not affect the OS rate (p≥0.05) (Figure 5).

## DISCUSSION

Uterine adenosarcoma comprises benign epithelial elements and malignant mesenchymal components that include a leaf-like architecture, periglandular condensation, mild/moderate cytologic atypia, and variable mitotic activity (2,3). These tumors generally have low malignant potential and a good prognosis following surgery. However, SOG is typically associated with a poor prognosis and recurrence (2–4). Additionally, although SOG is typically associated with a high-grade morphology, it can also be observed in low-grade adenosarcoma. Therefore, classifying adenosarcomas as high- or low-grade is useful for predicting tumor behavior. Soslow and Longacre first proposed that high-grade adenosarcomas have an aggressive course (8). Hodgson et al. reported that high-grade adenosarcoma was frequently associated with large tumor size and a high mitotic index (5). In this study, we found a significant association between high-grade adenosarcoma and the presence of SOG, lymphovascular invasion, heterologous elements, and a high mitotic count but no relation with FIGO stage or myometrial invasion. Additionally, we found that a high-grade morphology had a significant impact on the DFS and OS rates.

The additional clinicopathological features of the tumors in our series were concordant with those of the previously reported studies. Similar to outcomes from the literature, we found that patients with SOG had significantly shorter DFS and OS rates than patients without SOG, while lymphovascular invasion and stage also had a significant impact on DFS and OS rates (6,17,18). Additionally, we found that mitotic count had a significant impact on DFS and OS rates in our study.

Several studies have described the immunohistochemical features of adenosarcoma. The mesenchymal component of adenosarcoma has a similar immunophenotype to low-grade endometrial stromal sarcoma. Both tumors express ER, PR, CD10, and WT1 (6,19). Additionally, low-grade stromal components without sarcomatous overgrowth have shown a higher percentage of ER and PR positivity compared to high-grade sarcomatous components in adenosarcoma (6,19,20). Furthermore, decreased CD10 expression in adenosarcoma with sarcomatous overgrowth was observed compared with classic adenosarcoma (6,19,20). In this study, we found that adenosarcomas with a high-grade morphology reflected lower ER, PR, and CD10 immunoreactivity than those with a low-grade morphology. (6,19,20). We also showed that ER and PR immunoreactivity were lower and weaker in adenosarcomas that had heterologous elements compared to adenosarcomas that had any heterologous elements. Additionally, the loss of ER and CD10 expression in the stromal component of uterine adenosarcoma had a significant impact on OS. The loss of CD10 expression, but not of ER and PR expression, had a significant impact on DFS.

p16 and cyclin D1 play a specific role in the regulation of the G1-to-S phase in cell cycles (12,13). The overexpression of cyclin D1 has been observed in various types of human malignancies, including uterine sarcomas (15). There is limited data regarding cyclin D1 immunoreactivity in uterine sarcomas. Cyclin D1 immunostaining was specifically observed in endometrial stromal sarcoma (ESS), particularly YWHAE-FAM22 rearranged ESS (16). However, cyclin D1 has also been expressed in undifferentiated endometrial sarcoma and leiomyosarcoma without the YWHAE-FAM22 rearrangement (16). Gallardo et al. studied cyclin D1 immunoreactivity in uterine adenosarcoma, carcinosarcoma, endometrial stromal tumors, endometrial polyps, and endometriosis (6). The authors found no differences between cyclin D1 immunoreactivity and uterine adenosarcoma or other lesions (6). Omi et al. have reported that 3 of 7 uterine adenosarcomas had cyclin D1 immunoreactivity (2 had a high-grade morphology and SOG, and one was a low-grade type without SOG) (7). Lee et al. have reported that 25 adenosarcomas (8 of which had SOG) had no cyclin D1 immunoreactivity (16). Sharma and Prachi reported that one adenosarcoma with SOG showed cyclin D1 positivity (21). In the present study, we found that five (45.5%) of 11 high-grade adenosarcomas (all had SOG) and 3 (33.3%) of 9 low-grade adenosarcomas exhibited cyclin D1 immunoreactivity; however, no significant association was found between cyclin D1 immunoreactivity and tumor grade, the presence of SOG, or other clinicopathological parameters. In addition, our study indicated that cyclin D1 is positively correlated with an unfavorable DFS rate but not with OS.

In current gynecological pathology practice, diffuse block-type p16 expression is a surrogate marker for human papillomavirus (HPV) infection in cervical lesions. Additionally, p16 positivity is linked to non-HPV-related mechanisms, and p16 immunoreactivity has also been examined in a few studies involving uterine adenosarcoma (6). Gallardo and Prat demonstrated weak p16 immunoreactivity in endometrial polyp and endometrial stromal sarcomas, moderate p16 immunoreactivity in uterine adenosarcoma, and strong p16 immunoreactivity in carcinosarcoma (6). In the current study, the vast majority of cases (80%) exhibited p16 immunoreactivity. Furthermore, all high-grade adenosarcomas showed p16 immunoreactivity, and a significant correlation was found between the two. As such, p16 immunoreactivity was critical for confirming the presence of a high-grade morphology. All recurrent tumors showed p16 immunoreactivity. However, we demonstrated that p16 immunoreactivity did not affect DFS or OS rates. The current study observed CD117 negativity in uterine adenosarcoma to be similar to that reported in studies on mesenchymal tumors of the uterus (19,22). In contrast, other studies have shown a variable frequency of CD117 immunoreactivity in uterine sarcoma, but no mutation has been indicated to date (23–25).

A mutation in one of the repair proteins will lead to impairment in the DNA MMR system. Mismatch repair deficiency giving rise to MSI and malignancy has been identified in various cancer types, including gynecological cancers (9–11). In existing studies, MSI was more frequently found in uterine carcinosarcoma compared with other uterine sarcomas including leiomyosarcoma, endometrial stromal sarcoma, and rhabdomyosarcoma (26–28). The MSI status of uterine adenosarcoma and its prognostic effects have been less frequently studied. Risinger et al. evaluated only one adenosarcoma case for MSI status, which was found to be microsatellite-stable (29). Hoang et al. reported 11 adenosarcomas that showed intact MMR protein expression (26). In contrast to these studies, we demonstrated that 20% of cases had MSI. All MSI tumors had MLH1 loss, and one tumor had PMS2 and MSH2 loss in addition to MLH1 loss. Microsatellite instability is also known to be prognostic within various cancer types. Some studies provide evidence that increasing microsatellite stability is positively correlated with survival time in various cancer types (30). However, the current study showed MSI had no impact on OS or DFS rates in uterine adenosarcoma patients.

There were some limitations to our study. First, due to the rarity of adenosarcoma and the single-center nature of this study, the number of patients included in our research was limited. Second, we evaluated ER, PR, CD10, p16, cyclin D1, and MMR protein expressions using TMA cores, which were prone to assessment limitations. Third, we detected protein expression only by immunohistochemistry. We could not perform polymerase chain reaction or a methylation test of the MLH1. Consequently, these results should be validated by additional multi-center studies using a larger patient cohort.

In conclusion, the current study aimed to highlight the prognostic clinicopathological and immunohistochemical features of adenosarcoma. p16 positivity, along with the loss of ER, PR, and CD10 expression, were predictors of a high-grade morphology. We identified a high-grade tumor, the presence of sarcomatous overgrowth, lymphovascular invasion, a high mitotic count, and the presence of heterologous elements as poor prognostic factors for patients with uterine adenosarcoma. Additionally, the current study showed that cyclin D1-positive tumors had high recurrence rates; however, no significant relationships were found between MSI and DFS or OS rates in patients with uterine adenosarcoma. Accordingly, immunohistological features, along with tumor grade, may be useful for predicting the behavior of a tumor. Additional studies are needed to enable accurate predictions of the effect of p16 and cyclin D1 expression and MSI in uterine adenosarcoma cases.

## Conflict of Interest

The authors declare no conflict of interest.
